# GABPA protects against gastric cancer deterioration via negatively regulating GPX1

**DOI:** 10.5937/jomb0-35445

**Published:** 2022-07-29

**Authors:** Binghua Yin, Bing Dong, Xiaohui Guo, Can Wang, Huazhi Huo

**Affiliations:** 1 Handan Central Hospital, CT Room, Handan, China; 2 Handan Central Hospital, Department of Gastroenterology, Handan, China; 3 Handan Central Hospital, Department of General Surgery, Handan, China

**Keywords:** GABPA, GPX1, gastric cancer, migration, GABPA, GPKS1, rak želuca, migracija

## Abstract

**Background:**

To explore the anti-cancer role of GABPA in the progression of gastric cancer (GC), and the underlying mechanism.

**Methods:**

Quantitative real-time polymerase chain reaction (qRT-PCR) was conducted to detect the expression pattern of GABPA in 45 pairs of GC and non-tumoral tissues. The relationship between GABPA expression and clinic pathological indicators of GC patients was analyzed. In AGS and SGC-7901 cells overexpressing GABPA, their migratory ability was determined by trans well and wound healing assay. The interaction between GABPA and its downstream target GPX1 was explored by dual-luciferase reporter assay, and their synergistical regulation on GC cell migration was finally elucidated.

**Results:**

GABPA was downregulated in GC tissues in comparison to normal ones. Low level of GABPA predicted high incidences of lymphatic and distant metastasis in GC. Overexpression of GABPA blocked AGS and SGC-7901 cells to migrate. GABPA could target GPX1 via the predicted binding site. GPX1 was upregulated in clinical samples of GC, and negatively correlated to GABPA level. The anticancer effect of GABPA on GC relied on the involvement of GPX1.

**Conclusions:**

GABPA is downregulated in GC samples, which can be utilized to predict GC metastasis. Serving as a tumor suppressor, GABPA blocks GC cells to migrate by targeting GPX1.

## Abstract (sr)

**Uvod:**

Cilj je bio da se istraži antikarcenogena uloga GABPA u progresiji raka želuca (GC) i osnovni mehanizam.

**Metode:**

Kvantitativna lančana reakcija polimeraze u realnom vremenu (kRT-PCR) je sprovedena da bi se otkrio obrazac ekspresije GABPA u 45 parova GC i netumorskih tkiva. Analiziran je odnos između ekspresije GABPA i kliničkopatoloških pokazatelja pacijenata sa GC. U ćelijama AGS i SGC-7901 koje prekomerno eksprimiraju GABPA, njihova migraciona sposobnost je određena testom transvella i zarastanja rana. Interakcija između GABPA i njegovog nizvodnog ciljanog GPKS1 istražena je testom sa dvostrukom luciferazom, a njihova sinergistička regulacija migracije GC ćelija je konačno razjašnjena.

**Rezultati:**

GABPA je smanjena u GC tkivima u poređenju sa normalnim. Nizak nivo GABPA predvi|a visoku incidencu limfnih i udaljenih metastaza u GC. Prekomerna ekspresija GABPA je blokirala AGS i SGC-7901 ćelije da migriraju. GABPA bi mogao da cilja GPKS1 preko predviđenog mesta vezivanja. GPKS1 je bio pojačan u kliničkim uzorcima GC i imao je negativnu korelaciju sa nivoom GABPA. Efekat GABPA protiv raka na GC oslanjao se na učešće GPKS1.

**Zaključak:**

GABPA je smanjena u GC uzorcima, što se može koristiti za predviđanje GC metastaza. Služeći kao supresor tumora, GABPA blokira GC ćelije da migriraju ciljajući GPKS1.

## Introduction

Gastric cancer (GC) is the number two killer of cancer death [1] [2]. Recently, a growing number of therapeutic strategies for GC have been developed and improved, especially novel targeted therapy. However, the 5-year survival of metastatic GC is not ideal [3] [4]. The study results demonstrated that GC cells are prone to metastasize through lymph nodes [4]. A large number of GC patients are in the progressive stage at the first time of clinical diagnosis, thus losing the best surgical opportunity [3] [4] [5]. Therefore, to clarify the mechanism of GC metastasis and actively explore new biomarkers are urgently to be solved [6] [7] [8].

GA-binding protein alpha (GABPA) encodes one of the three GA-binding protein transcription factor subunits, serving as a DNA-binding subunit. Since this subunit has a common feature to that encoding the nuclear respiratory factor 2 gene, it may participate in the activation of cytochrome oxidase expression and nuclear control of mitochondrial function [9] [10]. GABPA also has a similar structure to that of the transcription factor E4TF1. Hence, it is involved in the expression of the adenovirus E4 gene [11]. Current evidences have proven the interaction between GABPA and several important transcription factors (i.e. HCFC1, SP1 and SP3), and its vital function in affecting pathways of interactions at neuromuscular junction and mitochondrial gene expression [12]. In multiple types of tumor cells, GABPA can selectively bind to the ETSdomain of the mutated telomerase reverse transcriptase (TERT) promoter and activate its transcription [13] [14]. Previous studies have already reported the involvement of GABPA in the progression of bladder cancer and breast cancer [14] [15]. Its potential influence in GC, however, is unclear.

This study aims to elucidate the role of GABPA in the malignant progression of GC and the molecular mechanism, which provides a novel target for individualized therapy.

## Materials and methods

### GC Samples

Forty-five GC patients with surgical resection in our hospital were retrospectively analyzed, and these patients did not have preoperative anti-cancer treatment, previous infection of Helicobacter Pylori (HP) was all positive as well as pathologically was confirmed as GC. Invasive GC tissues and adjacent normal tissues were harvested during subtotal gastrectomy and stored in liquid nitrogen. Follow-up of each GC patient through telephone and outpatient review was conducted after discharge, including physical conditions, clinical symptoms and signs, and imaging examinations. In addition, the patients with other malignancies; mental disease; myocardial infarction; heart failure or other chronic diseases, or those previously exposed to radioactive rays were excluded. This investigation was approved by the research Ethics Committee of Handan Central Hospital and complied with the Helsinki Declaration. Informed consent was obtained from patients.

### Cell lines and reagents

GC cell lines (AGS, BGC-823, SGC-7901) and the human gastric mucosal epithelial cell line (GES-1) were provided by American Type Culture Collection (ATCC) (Manassas, VA, USA). Cells were cultivated in Dulbecco's Modified Eagle's Medium (DMEM) (Gibco, Rockville, MD, USA) containing 10% fetal bovine serum (FBS) (Gibco, Rockville, MD, USA), 100 U/mL penicillin and 100 μg/mL streptomycin. Cell passage was conducted at 90% confluence using 1×tyrpsin containing EDTA (ethylenediaminetetraacetic acid).

### Transfection

Transfection plasmids were synthesized by GenePharma (Shanghai, China). Cells were cultured to 30-50% density in a 6-well plate, and transfected using Lipofectamine 2000 (Invitrogen, Carlsbad, CA, USA). After 48 h cell transfection, cells were collected for verifying transfection efficacy and functional experiments.

### Transwell migration assay

Cell suspension was prepared at 5×10^5^ cells/mL. 200 μL of suspension and 700 μL of medium containing 20% FBS was respectively added on the top and bottom of a transwell insert and cultured for 48 h. Migratory cells on the bottom were induced with methanol for 15 min, 0.2% crystal violet for 20 min and captured using a microscope. Five random fields per sample were selected for capturing and counting migratory cells.

### Wound healing assay

Cell suspension in serum-free medium was prepared at 5×10^5^/mL and implanted in 6-well plates. Cells were cultivated to 90% density, followed by creating an artificial scratch using a sterilized pipette tip. Cells were washed in phosphate-buffered saline (PBS) for 2-3 times and cultured in the medium containing 1% FBS. 24 hours later, wound closure percentage was calculated.

### Quantitative real-time polymerase chain reaction (qRT-PCR)

Cells were lysed using TRIzol reagent (Invi trogen, Carlsbad, CA, USA) for isolating RNAs. Qualified RNAs were reversely transcribed into complementary deoxyribose nucleic acids (cDNAs) using AMV reverse transcription kit (TaKaRa, Otsu, Shiga, Japan), followed by qRT-PCR using SYBR® Premix Ex Taq™ (TaKaRa, Otsu, Shiga, Japan). Glyceraldehyde 3-phosphate dehydrogenase (GAPDH) was the internal reference. Each sample was performed in triplicate, and relative level was calculated by 2^-ΔΔCt^. Primer se quences were as follows. GABPA: Forward: 5'-GG A GGAAGT GGAGG GA-CT GA-3', reverse: 5'-GCTTACACATTCAGCTGGCG-3'; GPX1: Forward: 5'-TA T CGAGAATGTGGCGTCCC-3', reverse: 5'-TCTTGGCGT T CTCCT GATGC-3'; GAPDH: forward: 5'-CCTGGCACCCAGCACAAT-3', reverse: 5'-TGCCGTAGGTGTCCCTTTG-3'.

### Western blot

Cells were lysed in radio immunoprecipitation assay (RIPA) (Beyotime, Shanghai, China) on ice for 15 min, and the mixture was centrifuged at 14000×g, 4 for 15 min. The concentration of cellular protein was determined by bicinchoninic acid (BCA) method (Beyotime, Shanghai, China). Protein samples with the adjusted same concentration were separated by sodium dodecyl sulphate-polyacrylamide gel electrophoresis (SDS-PAGE) and loaded on polyvinylidene difluoride (PVDF) membrane (Millipore, Billerica, MA, USA). The membrane was cut into small pieces according to the molecular size and blocked in 5% skim milk for 2 h. They were incubated with primary and secondary antibodies, followed by band exposure and grey value analyses.

### Dual-Luciferase reporter assay

Wild-type and mutant-type GABPA vectors were synthesized based on bioinformatics screening on the binding site to GPX1. They were co-transfected in HEK293T cells with either pcDNA-NC or pcDNA-GPX1 for 48 h. Luciferase activity was finally measured in a standard method (Promega, Madison, WI, USA).

### Statistical analysis

GraphPad Prism 5 V5.01 (La Jolla, CA, USA) was used for statistical analyses and data were expressed as mean ± standard deviation. Differences between groups were compared by the *t*-test. The relationship between GABPA expression and clinicopathological indicators of GC patients was analyzed by Chi-square test. P<0.05 was considered as statistically significant.

## Results

### GABPA was lowly expressed in GC

**Figure 1:**
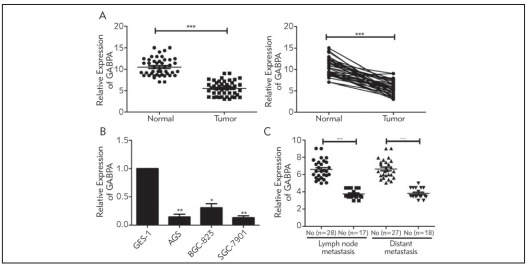
Proportion of critical values of the hospital during 2019 (before optimization) and 2020 (after optimization)

Forty-five cases of GC and paired adjacent normal tissues were collected in our center. It is shown that GABPA was downregulated in GC tissues (Figure 1A). In addition, GABPA was lowly expressed in GC cells in comparison to the human gastric mucosal epithelial cells (Figure 1B). It is speculated that GABPA may be a tumor suppressor gene involved in GC. We subsequently analyzed clinicopathological indicators of GC patients based on their GABPA levels. It is demonstrated that GABPA was closely linked to the incidences of lymphatic and distant metastasis in GC patients (Table 1). As expected, GC cases with lymphatic or distant metastasis expressed lower level of GABPA than nonmetastatic ones (Figure 1C).

**Table 1:** Association of GABPA expression with clinicopathologic characteristics of gastric cancer

Parameters	Number of cases	GABPA expression		P-value
		High (n=23)	Low (n=22)	
Age (years)				0.181
<60	23	14	9	
≥60	22	9	13	
Gender				0.295
Male	22	13	9	
Female	23	10	13	
T stage				0.182
T1-T2	25	15	10	
T3-T4	20	8	12	
Lymph node metastasis				0.023
No	28	18	10	
Yes	17	5	12	
Distance metastasis				0.011
No	17	18	9	
Yes	18	5	13	

### Knockdown of GABPA blocked GC to migrate

**Figure 2:**
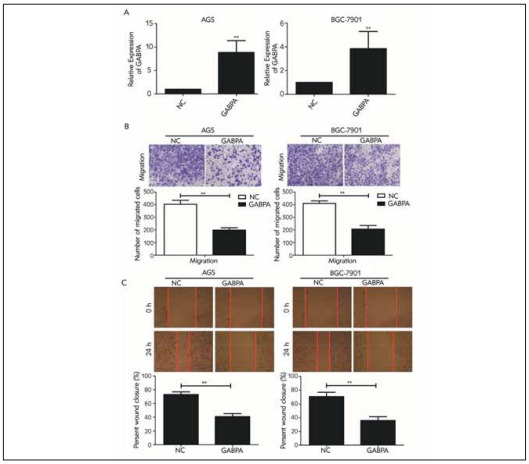
Proportion of critical values of the hospital during 2019 (before optimization) and 2020 (after optimization)

To explore the biological functions of GABPA in GC, pcDNA-GABPA was generated and transfection of pcDNA-GABPA effectively upregulated GABPA in AGS and SGC-7901 cells (Figure 2A). Later, overexpression of GABPA was identified to reduce migratory cell number and wound closure percentage in GC cells, suggesting the attenuated migratory potential (Figure 2B, Figure 2C).

### GABPA was bound to GPX1

**Figure 3:**
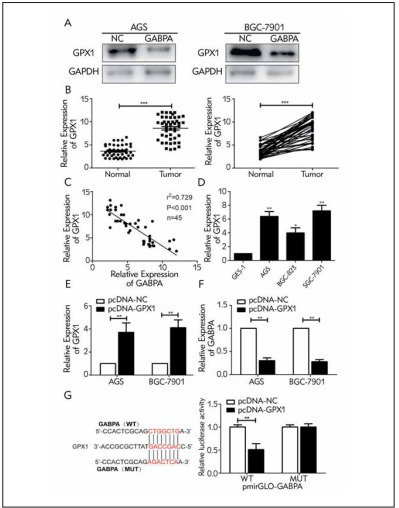
3GABPA was bound to GPX1. (A) Protein level of GPX1 in AGS and SGC-7901 cells overexpressing GABPA; (B) Differential levels of GPX1 in GC and adjacent normal tissues; (C) A negative correlation between mRNA levels of GPX1 and GABPA in GC tissues; (D) GPX1 levels in GC cell lines; (E) Transfection efficacy of pcDNA-GPX1; (F) GABPA level in AGS and SGC-7901 cells overexpressing GPX1; (G) Binding relationship between GABPA and GPX1. *P< 0.05, ***P< 0.001

Using online bioinformatics tools, it is predicted that GABPA may bind to GPX1. Western blot analysis showed that protein level of GPX1 was markedly downregulated in AGS and SGC-7901 cells overexpressing GABPA (Figure 3A). GPX1 was detected to be highly expressed in GC tissues and cell lines (Figure 3B, Figure 3D). It was negatively correlated to mRNA level of GABPA in clinical samples of GC (Figure 3C). Subsequently, transfection efficacy of pcDNA-GPX1 was examined (Figure 3E). Overexpression of GPX1 downregulated GABPA in GC cells, further supporting their negative correlation (Figure 3F). Dual-luciferase reporter assay revealed a decline of luciferase activity in the wild-type GABPA vector after overexpression of GPX1. Nevertheless, GPX1 could not affect luciferase activity in the mutant-type one (Figure 3G). As a result, we have proven that GPX1 could be targeted by GABPA through the predicted binding site.

### GABPA and GPX1 synergistically regulated GC migration

**Figure 4:**
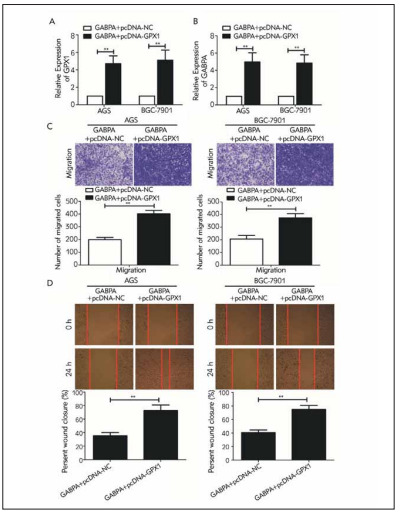
GABPA and GPX1 synergistically regulated GC migration. (A) GPX1 level in AGS and SGC-7901 cells co-overexpressing GABPA and GPX1; (B) GABPA level in AGS and SGC-7901 cells co-overexpressing GABPA and GPX1; (C) Migration in AGS and SGC-7901 cells co-overexpressing GABPA and GPX1 (magnification 20×); (D) Wound closure in AGS and SGC-7901 cellsco-overexpressing GABPA and GPX1 (magnification 20×). *P< 0.05, **P< 0.01.

To further uncover the synergistical regulation of GABPA and GPX1 on GC, we co-transfected pcDNA-GABPA and pcDNA-GPX1 in AGS and SGC-7901 cells. Compared with those overexpressing GABPA, both GPX1 and GABPA levels were much higher in AGS and SGC-7901 cells co-overexpressing GABPA and GPX1 (Figure 4A, Figure 4B). Co-overexpression of GABPA and GPX1 enhanced migratory potential in GC cells with solely overexpression of GABPA (Figure 4C, Figure 4D).

## Discussion

Globally, GC is a severe cancer that has high incidence and mortality, and is also one of the digestive system in our country [1] [2] [3]. Epidemiological investigations have proposed that the carcinogenesis of GC involves environmental, biological, genetic and epigenetic factors [3] [4]. Microarray analysis is an effective technology to identify differentially expressed genes in the profile of cancer. These abnormally activated or inactivated genes are potential molecular biomarkers for screening or predicting the progression of human cancers [5] [6] [7].

Previous study reported that GABPA could inhibit the metastasis of papillary thyroid carcinoma through regulating DICER1 [16]. In addition, another study revealed that it served as a novel biomarker for the prognosis of hepatocellular carcinoma since GABPA was able to block the migration of cancer cells by regulating E-cadherin [17]. However, the function of GABPA in HCC is not clear. Our findings showed that GABPA was downregulated in GC tissues, compared to adjacent normal ones. Low level of GABPA predicted high incidences of lymphatic and distant metastasis in GC patients. Thus, it is speculated that GABPA could be a tumor suppressor involved in the progression of GC. Later, a series of functional experiments identified that overexpressing GABPA, the migratory potential was markedly inhibited in AGS and SGC-7901 cells.

We thereafter verified that GPX1 was the downstream target binding GABPA by miRDB starbase. GPXs (glutathione peroxides) are vital antioxidant enzymes in the body, which are responsible for eliminating ROS [18]. GPX1 is the most common antioxidant enzyme in the GPX family. It is widely present in human cells, and has antioxidant and detoxifying effects [19] [20]. Protein level of GPX1 was found to be downregulated by overexpression of GABPA in GC cell lines. In addition, we also found that GPX1 was upregulated and negatively correlated to GABPA level in GC tissues. Furthermore, the rescue experiments identified the migration ability of GPX1 to reverse the regulatory effect of GABPA on GC cell lines. Taken together, GABPA was a tumor suppressor that inhibited migratory potential in GC through negatively regulating GPX1, which could be utilized in the clinical targeted therapy of GC.

## Conclusions

GABPA is downregulated in GC samples, which can be utilized to predict GC metastasis. Serving as a tumor suppressor, GABPA blocks GC cells to migrate by targeting GPX1.

## Dodatak

### Conflict of interest statement

All the authors declare that they have no conflictof interest in this work.

### List of Abbreviations

Gastric cancer (GC);<br>GA-binding protein alpha (GABPA);<br>telomerase reverse transcriptase (TERT);<br>Helicobacter Pylori (HP);<br>gastric mucosal epithelial cell line (GES-1);<br>American Type Culture Collection (ATCC);<br>Dulbecco’s Modified Eagle’s Medium (DMEM);<br>fetal bovine serum (FBS);<br>ethylenediaminete-traacetic acid (EDTA); <br>phosphate-buffered saline (PBS);<br>Quantitative Real-Time Polymerase Chain Reaction (qRT-PCR);<br>complementary deoxyribose nucleic acids (cDNAs);<br>Glyceraldehyde 3-phosphate dehydrogenase (GAPDH);<br>radio immunoprecipitationassay (RIPA);<br>bicinchoninic acid (BCA);<br>sodium dodecyl sulphate-polyacrylamide gel electrophoresis (SDS-PAGE);<br>polyvinylidene difluoride (PVDF);<br>glutathione peroxides (GPXs).
